# Synergies and Entanglement in Secondary Cell Wall Development and Abiotic Stress Response in Trees

**DOI:** 10.3389/fpls.2021.639769

**Published:** 2021-03-19

**Authors:** Heather D. Coleman, Amy M. Brunner, Chung-Jui Tsai

**Affiliations:** ^1^Department of Biology, Syracuse University, Syracuse, NY, United States; ^2^Department of Forest Resources and Environmental Conservation, Virginia Tech, Blacksburg, VA, United States; ^3^Department of Plant Biology, University of Georgia, Athens, GA, United States; ^4^Department of Genetics, University of Georgia, Athens, GA, United States; ^5^Warnell School of Forestry and Natural Resources, University of Georgia, Athens, GA, United States

**Keywords:** abiotic stress, secondary cell wall, *Populus*, drought, nutrient stress, gene duplication

## Abstract

A major challenge for sustainable food, fuel, and fiber production is simultaneous genetic improvement of yield, biomass quality, and resilience to episodic environmental stress and climate change. For *Populus* and other forest trees, quality traits involve alterations in the secondary cell wall (SCW) of wood for traditional uses, as well as for a growing diversity of biofuels and bioproducts. Alterations in wood properties that are desirable for specific end uses can have negative effects on growth and stress tolerance. Understanding of the diverse roles of SCW genes is necessary for the genetic improvement of fast-growing, short-rotation trees that face perennial challenges in their growth and development. Here, we review recent progress into the synergies and antagonisms of SCW development and abiotic stress responses, particularly, the roles of transcription factors, SCW biogenesis genes, and paralog evolution.

## Introduction

The plant secondary cell wall (SCW) plays important roles. In the stem, it participates in structure, form, and function, as a part of the water transport system. Throughout the plant, it is an important part of the defense system, as a barrier to attack, and in systematic response to biotic and abiotic stress. The cell wall acts as the first line of defense, and cell wall integrity sensing and maintenance are tightly integrated with biotic and abiotic stress signaling (Bacete et al., 2018). Cell wall plasticity is key to a plant’s capacity to adjust to environmental conditions, such as water and nutrient availability, and adapt to specific climates (Landi and Esposito, 2017; Lee et al., 2017). The stress response role of the cell wall has been previously reviewed, particularly with regards to cell wall integrity as a mechanism for sensing and responding to stress (Novakovic et al., 2018; Vaahtera et al., 2019; Anderson and Kieber, 2020). Here, we focus on trees, highlighting examples of regulatory and SCW metabolism genes that indicate both synergy and antagonism in achieving multiple goals of improved stress resilience, biomass yield, and biomass quality.

The SCW consists of a complex network of cellulose, hemicellulose, and lignin (reviewed by Kumar et al., 2016; Meents et al., 2018; Zhong et al., 2019). Prior to maturity, production of this network is influenced by external factors. These interactions and their effect on biomass yield and quality traits are especially complex in trees where harvested wood is the result of a multi-year history, from 2- to 3-year coppice cycles to decades of genotype × environment interactions. Optimum growth depends on developmental and physiological transitions being appropriately timed for the local climate (Cooke et al., 2012; Brunner et al., 2017). In temperate and boreal zones, this requires preparation for the cold and dehydration stresses of winter and subsequent reversal to allow resumption of growth with transitions occurring at seasonal times that avoid frost injury and optimally capture resources to support growth. Although photoperiod, prolonged chilling temperatures and accumulated exposure to warm temperature are primary signals for key phenological changes, other environmental factors modulate the timing and rate of these transitions (Cooke et al., 2012; Ding and Nilsson, 2016; Brunner et al., 2017; Maurya and Bhalerao, 2017). In temperate regions, water stress generally increases in mid to late summer, a trend thought to drive the transition from large vessel earlywood to dense, small vessel latewood (Plomion et al., 2001). In seasonally dry tropical climates, the intra-annual development of some tree taxa is characterized by distinct periods of rest and rapid shoot growth. Temperate taxa such as *Populus*, *Betula*, and many *Salix* species are free growing, a major factor for suitability as short rotation woody biomass crops. Their shoots have the capacity to continue to grow until a critical daylength threshold occurs; this capacity is limited by other factors, such as water and nutrient availability. Thus, achieving optimal woody biomass yield and quality requires increased understanding of the interplay among primary determinates of seasonal transitions and more episodic or site-specific stresses.

Not surprisingly, manipulation of SCW biosynthesis can result in widespread changes in both the metabolome and transcriptome that might lead to negative effects on growth and development (Xie et al., 2018b; Vanholme et al., 2019). Frequently, there are indications that altering SCW biosynthesis genes can have a direct or indirect impact on the plant’s response to abiotic stress. There is also evidence that sub-functionalization or neo-functionalization of cell wall biosynthesis gene duplicates can have an impact on the relationship between stress-resistance and cell wall structure. Here, we examine the interaction between the SCW and abiotic stress responses, highlighting examples of transcription factors (TFs) and SCW biogenesis genes that directly impact both biomass and stress response, as well as the sub-/neo-functionalization of SCW biosynthesis gene paralogs in the plant response to abiotic stress.

## Transcriptional Regulation of SCW and Abiotic Stress

The intricate regulation of plant growth and stress response is directed in part by a large number of TFs, including MYB and NAC family members (Wilkins et al., 2009; Hu et al., 2010; Ye and Zhong, 2015; Chen et al., 2019). Dual roles of some TFs have been reported in both SCW formation and abiotic stress responses.

An ortholog of *Arabidopsis MYB46* (Zhong et al., 2007) from *Betula platyphylla* was overexpressed and silenced in birch (Guo et al., 2017). Overexpression lines showed improved growth under both salt and osmotic stress, while silenced lines were reduced in growth including above and below ground biomass, as well as chlorophyll content. Overexpression lines had increased levels of proline and reactive oxygen species (ROS) scavenging, attributed to increased expression of *Δ1-pyrroline-5-carboxylate synthetase* (*P5CS*), *superoxide dismutase* (*SOD*), and *peroxidase* (*POD*) genes. The overexpressing lines had increased lignin and cellulose levels and thicker fiber cell walls, but decreased hemicellulose relative to WT; silenced lines showed the opposite pattern. This was attributed to alterations in expression of a suite of lignin biosynthesis genes as well as *cellulose synthases* (*CesAs*), with increased expression in the overexpression lines, but reduced expression in silenced lines. Hemicellulose-related genes displayed the opposite pattern. ChIP-PCR supported interactions between BpMYB46 and promoters of the above-mentioned genes involved in ROS, proline, and SCW biosynthesis.

AtMYB61 induces ectopic lignification and dark photomorphogenesis in *Arabidopsis* (Newman et al., 2004). *AtMYB61* is also expressed in guard cells and its mis-expression has a direct impact on stomatal apertures, smaller in overexpressors and larger in knockout mutants relative to WT (Liang et al., 2005). The *Populus* ortholog PtoMYB170 positively regulates lignin biosynthetic genes, as evidenced by enhanced lignin deposition in PtoMYB170-overexpressing plants and reduced lignification in CRISPR-knockout (KO) lines (Xu et al., 2017). *PtoMYB170* is specifically expressed in guard cells and confers enhanced drought tolerance when overexpressed in *Arabidopsis* (Xu et al., 2017). This suggests that the dual functionality in SCW biogenesis and abiotic stress responses is evolutionarily conserved.

A dual role has also been reported for NAC secondary wall thickening promoting factor/secondary wall-associated NAC domain protein (NST/SND) orthologs in the regulation of SCW formation and abiotic stress resistance in both *Arabidopsis* and birch. *Arabidopsis snd1* mutants with impaired fiber SCW biogenesis (Zhong et al., 2006) were also shown to have reduced survival rate under salt stress (Jeong et al. 2018). Mutant lines had increased levels of ABA, which lends strength to the model that SND1 positively regulates MYB46 and lignin biosynthesis, and negatively regulates ABA signaling and biosynthesis (Jeong et al., 2018).

Hu et al. (2019) characterized the AtSND1 ortholog BpNAC012 in birch. BpNAC012 was expressed predominantly in stems and its expression in leaves increased in plants exposed to salt, osmotic, and drought stress. Silencing of BpNAC012 resulted in thinner fiber walls. While the cell wall thickness was unchanged in overexpression lines, these lines produced more biomass and were more tolerant to salt and osmotic stress, attributed to increased expression of *P5CS1* and *P5CS2* and increased SOD and POD activities. Multiple assays demonstrated interactions between BpNAC012 and the promoters of abiotic stress-responsive (SOD and POD) genes, as well as known lignin, cellulose, and hemicellulose biosynthesis genes and additional SCW TFs. The authors hypothesize a model in which BpNAC012 binds to the core sequence CGT[G/A] in regulation of genes associated with abiotic stress and binds to the SNBE site in regulation of genes involved in SCW biosynthesis (Hu et al., 2019).

Additional examples of transcriptional co-regulation of growth, defense, and lignification in herbaceous species are discussed in a recent review (Xie et al., 2018b). In trees, the dual role of TFs in regulation of SCW formation and abiotic stress resistance is likely to involve responses to seasonal signals and dormancy-growth transitions, as well as reactions to sporadic stress events.

## Roles of SCW Biogenesis Genes in Abiotic Stress Responses

The effects of abiotic stresses on SCW biogenesis and wood formation have been covered in other reviews (Moura et al., 2010; Houston et al., 2016; Camargo et al., 2019; Eckert et al., 2019). Although few studies have investigated the role of SCW synthesis genes in stress responses in trees, many transcriptomic studies suggest roles for SCW biosynthesis in both seasonal adaptations and abiotic stress responses, with consequential effects on biomass utilization (Fox et al., 2017; Ployet et al., 2017; Wildhagen et al., 2017; Jokipii-Lukkari et al., 2018). For instance, drought-acclimated *Populus nigra* showed the expected reduction of cambial growth, with an unexpected increase of saccharification potential (Wildhagen et al., 2017). The increased sugar release was unrelated to lignin content but instead, was strongly associated with cell wall matrix polysaccharide biosynthesis and modification, based on gene coexpression network analysis (Wildhagen et al., 2017). Thus, besides well-documented effects of environmental stresses on lignin traits (Moura et al., 2010), the sensitivity of SCW polysaccharide biosynthesis to abiotic stresses also warrants attention.

A few studies show altered expression of stress-related genes in transgenics with modified wood characteristics. For instance, lignin-deficient poplars exhibited transcriptome reprogramming of genes associated with not only cell wall biogenesis and remodeling, but also ROS metabolism, detoxification, and response to various stimuli (Tsai et al., 2020). In particular, genes involved in the glutathione-ascorbate cycle, sulfate assimilation, and cadmium response were upregulated in lignin-reduced poplars. The patterns are in agreement with reported responses of cadmium-exposed plants, including poplars (Herbette et al., 2006; Van De Mortel et al., 2008; Elobeid et al., 2011; Ding et al., 2017), supporting a link between lignification and heavy metal-elicited oxidative stress responses.

Another study investigated tubulin genes encoding components of cortical microtubules that have long been thought to direct cellulose microfibril deposition during cell wall biogenesis (Baskin, 2001). Consistent with this role, several tubulin genes are among the most abundant transcripts in SCW-rich xylem (Hu et al., 2016). However, manipulation of tubulin genes can be lethal or result in abnormal development (Anthony et al., 1999; Burk et al., 2006; Ishida et al., 2007). In poplar, constitutive expression of xylem-biased tubulins led to abnormal vascular development, and plant regeneration was achieved only with post-translational modification mimics of tubulins (Swamy et al., 2015). Those plants showed tissue-dependent tubulin transgene expression, much higher in leaves than xylem, opposite to the expression of endogenous tubulins. No differences in major SCW constituents were detected in transgenic wood; however, extractability of lignin-bound pectin and xylan polysaccharides was increased, as was expression of genes encoding cell wall-modifying enzymes (Swamy et al., 2015). The authors suggest an association between pectin, xylan, and lignin during early stage of SCW biogenesis that is sensitive to subtle tubulin perturbation. In transgenic leaves with elevated expression of tubulin transgenes, pectin levels increased, while expression and activity of pectin methylesterase were reduced (Harding et al., 2018). Transgenic leaves also exhibited altered stomatal behavior, with delayed opening in response to light and delayed closure in response to drought (Swamy et al., 2015), consistent with microtubule involvement in guard cell dynamics. These studies add to the functional multiplicity of tubulins and microtubules in different phases of cell wall biogenesis, associated with both cellulosic and non-cellulosic polysaccharide assembly, and impacting both wood formation and stress responses.

Manipulation of SCW genes can cause tradeoffs between stress resistance and growth. Xyloglucan endotransglycosylase/hydrolase (XTH) acts in the relaxation of the cell wall, which is key in cell expansion during normal growth, and in cell wall remodeling during stress. XTH has been shown to allow or restrict cell wall expansion (Takeda et al., 2002), and to respond to drought stress (Iurlaro et al., 2016). In poplar, PtoXTH27 and PtoXTH34 were indicated to play a role in osmotic stress responses (Jiang et al., 2020).

Given the role of xylem in water transport (Rodriguez-Zaccaro and Groover, 2019), it is not surprising that alterations in wood composition often result in reduced water transport (Kitin et al., 2010). In *Arabidopsis*, this can be mitigated by restoring expression of SCW-related genes in vessels (De Meester et al., 2018). Hence, it will be interesting to test whether similar strategies can be an effective in improving wood quality and yield without negative effects on abiotic stress resilience.

## Sub-Functionalization of Duplicated Genes

**Figure 1:**
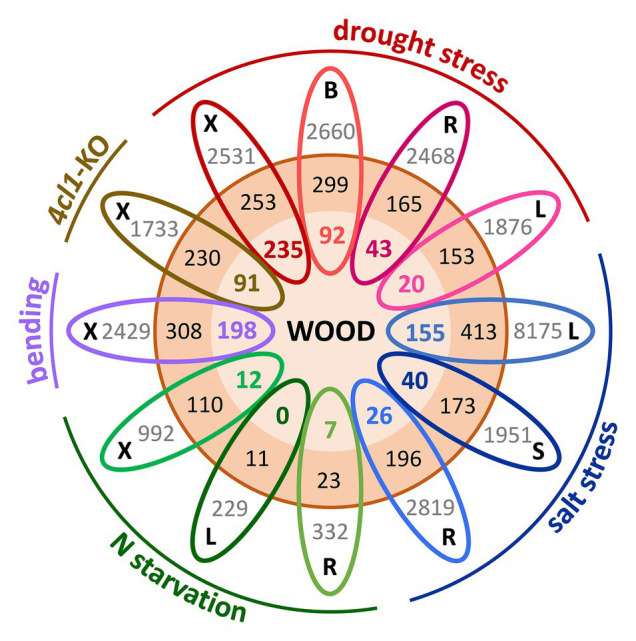
Gene expression divergence in response to abiotic stress among *Populus* wood-expressed paralogs. Salicoid duplicates (3,428 genes or 1,714 paralogs pairs, orange circle) showing expression correlations of ≥0.75 across the secondary stem (Sundell et al., 2017) were interrogated for their responsiveness to different perturbations, including nitrogen (N) starvation in roots, leaves (Luo et al., 2015), and xylem (Lu et al., 2019), tension wood induction by bending (Swamy et al., 2015), drought-stressed xylem, bark, roots, and leaves (Xue et al., 2016), xylem of lignin-deficient trees (4CL1-KO; Tsai et al., 2020), and salt-stressed leaves, stem, and roots (Yao et al., 2020). Each oval represents one tissue (B, bark; L, leaf; R, root; S, stem; and X, xylem) from a given experiment, color-coded by perturbation type. Gray values indicate the total number of differentially expressed genes for the specific tissue-stress combination as reported in the source paper. Boldfaced color values (inner orange circle) indicate wood-expressed paralogs gene pairs where both members of the pair were differentially expressed in response to the indicated stress, whereas black values (outer orange circle) denote the number of cases where only one paralog of a pair showed differential expression. For clarity of illustration, each stress dataset was compared with the wood paralogs individually without considering overlapping gene responses across tissues or stress conditions.

A largely unexplored area is the contribution of gene duplication and evolution to the integration or separation of genetic pathways involved in growth, stress resilience, and wood development. Members of the Salicaceae share a relatively recent WGD estimated to have occurred ~60 million years ago, and *Populus* retains ~8,000 Salicoid duplicate gene pairs (Tuskan et al., 2006; Rodgers-Melnick et al., 2012; Dai et al., 2014). Evidence of paralog regulatory divergence can be inferred from the growing wealth of RNA-seq datasets. Spatially-detailed expression profiling of the poplar secondary stem showed that Salicoid duplicates with peak expression during SCW deposition tended to exhibit highly-similar profiles, suggesting that many SCW-associated paralogs have functionally redundant roles (Sundell et al., 2017). However, increasing evidence supports a role for WGD in plant environmental adaptation (Wu et al., 2020); thus, we compared published transcriptomic studies for evidence that paralogs showing highly-correlated expression in the *Populus* woody stem (Sundell et al., 2017) exhibit regulatory divergence in response to different abiotic stresses (Luo et al., 2015; Swamy et al., 2015; Xue et al., 2016; Lu et al., 2019; Yao et al., 2020), as well as to SCW modification (Tsai et al., 2020). Strikingly, in all these stress-tissue combinations, only one member of these paralog pairs was differentially expressed in response to the stress more often than both paralogs (Figure 1). Examples of functional diversification of gene duplicates with regards to SCW synthesis are provided below. With increasing efforts in functional characterization of gene duplicates, the expectation is that neo-/sub-functionalization of wood-expressed paralogs will continue to be identified as one of the mechanisms for tree adaptation to varying environment.

One example is the poplar *5-enolpyruvylshikimiate 3-phosphate synthase* duplicate (Xie et al., 2018a); one encoding a classic EPSP synthase (PtrEPSP-SY) of the shikimate pathway, and the other harboring an extended N terminus with a helix-turn-helix DNA-binding motif (PtrEPSP-TF) with xylem-biased expression. Using linkage-disequilibrium based associate mapping, PtrEPSP-TF was found to exhibit associations with lignin content and syringyl-to-guaiacyl (S/G) ratio (Xie et al., 2018a). *PtrEPSP-TF* overexpression induces ectopic lignin and flavonoid biosynthesis through transcriptional repression of a hAT transposase PtrhAT. PtrhAT represses PtrMYB021, a MYB46 ortholog that regulates biosynthesis of SCW components, including lignin, cellulose, and xylan (Xie et al., 2018a). Neo-functionalization of a primary (shikimate) biosynthetic pathway gene with an additional role in transcriptional regulation of downstream secondary (phenylpropanoid) pathways represents an example of protein moonlighting conferring enhanced fitness of complex organisms (Copley, 2014).

A second example is the poplar paralogs of AtMYB61 involved in regulation of lignin biosynthesis and stomatal aperture noted above. While PtoMYB170 exhibits conserved dual functionality, guard cell expression was not detected for its Salicoid duplicate *PtoMYB216* (Xu et al., 2017). In this case, sub-functionalization might have resulted in more specific involvement of the poplar MYB216 in SCW biogenesis (Tian et al., 2013; Wei et al., 2019).

Another example concerns the poplar *4-coumarate:CoA ligase* (*4CL*) duplicates. In poplar, *4CL1* normally comprises ~90% of xylem *4CL* transcripts and encodes the predominant isoform involved in lignin biosynthesis (Hu et al., 1999; Voelker et al., 2010). *4CL1*-knockout led to ~20% lignin reduction and uniform wood discoloration (Zhou et al., 2015). Knockout of its Salicoid duplicate *4CL5*, the only other xylem-expressed *4CL* gene, has no effect on lignin accrual, suggesting a conditional role (Tsai et al., 2020). Nonetheless, the *4cl1* mutants maintain ~80% WT lignin levels, which must be sustained by *4CL5*. *4CL5* expression was not significantly changed in the *4cl1* mutants; however, *caffeoylshikimate esterase1* (*CSE1*) involved in caffeate biosynthesis and *caffeoyl-CoA O-methyltransferase1* (*CCoAOMT1*) that acts downstream of 4CL product caffeoyl-CoA were upregulated (Tsai et al., 2020). In contrast, the S lignin-specific *ferulate/coniferaldehyde 5-hydroxylases* were downregulated. These, along with elevated levels of caffeic acid in the mutant xylem hint at a novel mechanism for *in vivo* enhancement of 4CL5 function to sustain G lignin biosynthesis at the expense of S lignin (Tsai et al., 2020).

The preferential reductions of S lignin in the *4cl1* poplars (Zhou et al., 2015) contrasts with maize, sorghum, *Arabidopsis*, and switchgrass mutants where *4CL*-knockout led to strong G lignin reductions (Saballos et al., 2008; Van Acker et al., 2013; Park et al., 2017; Xiong et al., 2019). The molecular responses also differ between poplar and *Arabidopsis* mutants, with the latter showing upregulation of early pathway genes *phenylalanine ammonia-lyase2*, *cinnamate 4-hydroxylase*, and *4-coumaroylshikimate 3'-hydroxylase* (Vanholme et al., 2012). Gene coexpression network modeling revealed distinct associations between Salicoid paralogs of *4CL1/4CL5*, *CSE1/CSE2*, and *CCoAOMT1/CCoAOMT2* duplicates, with *4CL5*, *CSE1* and *CCoAOMT1* belonging to the same coexpression module in the *4cl1* mutant network (Tsai et al., 2020). The data provide evidence for coordinated subfunctionalization of multiple gene duplicates in the lignin pathway with conditional roles that may be key for lineage-specific adaptation.

## Conclusion

To meet the grand challenge for sustainable food, fuel, and fiber under changing climate requires a holistic understanding of diverse roles of SCW genes during plant growth, development, and interactions with the environment. Expanding functional characterization efforts promise to provide additional insights into many of the hidden/conditional roles. This is especially important for woody perennials with a rich repertoire of gene duplicates, many of which likely have evolved *via* sub-/neo-functionalization. The specificity of CRISPR genome editing allows the dissection of functional redundancy vs. specificity of gene duplicates, and the targeted selection of genes and gene duplicates to better understand connections between SCW formation and abiotic stress resistance.

The necessity of exploring the largely unexamined, but clear intersectional implications of SCW development and abiotic stress responses, particularly in the face of changing climate, is clear. Trees present the challenge of integrating multi-year growth accumulation with recurring seasonal phenology and episodic stress events. Reflecting this diversity of environmental interactions, poplar transgenics with altered expression of lignin biosynthesis genes have shown phenotypic differences between greenhouse and field studies (reviewed in Chanoca et al., 2019). Although transgenic tree responses to stress and seasonal cues in controlled conditions provides insight, more field studies are needed to delineate gene functions in trees, and to advance genetic engineering for simultaneous improvement of wood yield, quality, and resilience to environmental stress and climate change.

## Author Contributions

All authors contributed equally to writing and editing of this manuscript.

### Conflict of Interest

The authors declare that the research was conducted in the absence of any commercial or financial relationships that could be construed as a potential conflict of interest.
